# Consumption of animal source foods and dietary diversity reduce stunting in children in Cambodia

**DOI:** 10.1186/1755-7682-6-29

**Published:** 2013-07-17

**Authors:** Chau Darapheak, Takehito Takano, Masashi Kizuki, Keiko Nakamura, Kaoruko Seino

**Affiliations:** 1 Section of Health Promotion, Graduate School of Medical and Dental Sciences, Tokyo Medical and Dental University, Bunkyo City, Tokyo, Japan; 2 Section of International Health and Medicine, Graduate School of Medical and Dental Sciences, Tokyo Medical and Dental University, Bunkyo city, Tokyo, Japan

**Keywords:** Nutrition status, Children under 5 years, Demographic and health survey, Food groups

## Abstract

**Background:**

Malnutrition in children is a major public health concern. This study aimed to determine the association between dietary diversity and stunting, underweight, wasting, and diarrhea and that between consumption of each specific food group and these nutritional and health outcomes among children.

**Methods:**

A nationally representative household survey of 6209 children aged 12 to 59 months was conducted in Cambodia. We examined the consumption of food in the 24 hours before the survey and stunting, underweight, wasting, and diarrhea that had occurred in the preceding 2 weeks. A food variety score (ranging from 0 to 9) was calculated to represent dietary diversity.

**Results:**

Stunting was negatively associated with dietary diversity (adjusted odd ratios [OR_adj_] 0.95, 95% confident interval [CI] 0.91-0.99, *P* = 0.01) after adjusting for socioeconomic and geographical factors. Consumption of animal source foods was associated with reduced risk of stunting (OR_adj_ 0.69, 95% CI 0.54-0.89, *P* < 0.01) and underweight (OR_adj_ 0.74, 95% CI 0.57-0.96, *P* = 0.03). On the other hand, the higher risk of diarrhea was significantly associated with consumption of milk products (OR_adj_ 1.46, 95% CI 1.10-1.92, *P* = 0.02) and it was significantly pronounced among children from the poorer households (OR_adj_ 1.85, 95% CI 1.17-2.93, *P* < 0.01).

**Conclusions:**

Consumption of a diverse diet was associated with a reduction in stunting. In addition to dietary diversity, animal source food was a protective factor of stunting and underweight. Consumption of milk products was associated with an increase in the risk of diarrhea, particularly among the poorer households. Both dietary diversity and specific food types are important considerations of dietary recommendation.

## Background

Malnutrition is a major public health concern and an underlying cause of morbidity and mortality, particularly among children younger than 5 years of age, and it is a contributing cause of more than one-third of all childhood deaths [1-3]. Stunting is an important marker of chronic malnutrition whereas underweight reflects a combination of chronic and acute malnutrition [4]. Wasting refers to low weight-for-height where a child is thin for his/her height, but not necessarily short. This is known as an indicator of acute malnutrition. Stunting and underweight in early life is associated with greater risk of infection and with numerous long-term, effects, including diminished physical capacity for work [5]. Wasted children have a 5–20 times higher risk of dying from common diseases such as diarrhea or pneumonia than normally nourished children [5].

To provide a clear focus and long-term direction for addressing malnutrition in children in Cambodia, the national health strategic was designed and launched in 2002 to reduce the prevalence of stunted, underweight, and wasted children in 2015 to be 22%, 23%, and 5%, respectively [6]. According to anthropometric measurements in 2010, based on the new World Health Organization (WHO) growth standards, prevalence of stunting, underweight, and wasting among children younger than 5 years of age remained high (40%, 28%, and 11%, respectively) [7]. Stunting, underweight, and wasting are still the major issues for child development and growth in Cambodia.

Diarrhea is the second leading cause of death among children younger than 5 years of age worldwide. It was reported that about 1.5 million, or approximately 1 in 5 childhood deaths, are due to diarrhea [8]. In Cambodia, it was reported that 17% of death among children younger than 5 years were related to diarrhea in 2005 [9]. Also, diarrhea was reported to be associated with an increased risk of child malnutrition [10,11]. These data indicate the serious effect of diarrhea on children’s health and especially on child malnutrition.

“The Infant and Young Child Feeding (IYCF) Model Chapter” which was issued by the WHO recommended introducing complementary foods for infants starting at the age of 6 months. Breastmilk needs to be complemented by other foods to ensure that all the infant’s nutrient needs are met, although, by 6 month age, breastmilk is the most hygienic food and it meets most of the required nutrients by infants [12]. In promoting improvement in children’s nutritional status, the IYCF model chapter recommended an appropriate feeding based on specific food groups and locally acceptable and affordable. Such complementary foods included staple foods, animal source foods, milk products, green leafy and/or orange-colored vegetables, pulses oils/fats, and seeds [12]. Although consumption of these seven food groups by children is promoted, the relation of each food group to the nutritional status of children younger than 5 years of age was not clearly demonstrated by previous studies.

Dietary diversity was reflected in the number of foods consumed across and within food groups over a specified time period. These numbers were used as key dimensions to ensure adequate intake of essential nutrients to promote good health and to reflect the concept of increasing the variety of foods. It also plays a role in identifying the food security of the households and communities [13,14]. However, the effects of dietary diversity on the nutritional status of children are still questioned.

Our study aimed at: (i) determining the association between dietary diversity and stunting, underweight, wasting, and diarrhea among Cambodian children aged 12 to 59 months; and (ii) further identifying the association between the consumption of specific food groups and these health outcomes, regardless of socioeconomic and geographic variables.

## Methods

### Data source

Our study used data from the Cambodia Demographic and Health Survey (CDHS) in 2005. The survey collected demographic, socioeconomic, nutrition and health data from a nationally representative sample of 14,243 households. The survey employed intensive interviewer trainings and standardized measurement tools and techniques.

### Sampling procedure

All 24 provinces in the country were divided into nineteen sampling domains: 14 of which corresponded to individual provinces and 5 of which corresponded to groups of provinces, to attain balance between the ability to provide estimates for all provinces and limiting the sample size at the same time. Each sampling domain was subdivided into urban and rural according to the definition of Cambodia General Population Census, and 38 sampling strata were created.

Samples were selected independently in every stratum, by a two-stage selection procedure. The sample frame was developed by the National Institute of Statistics and consisted of 13,505 villages. In the first stage, 557 villages were selected with probability proportional to the number of households residing in the village. A listing of all the households was drawn up in each of the 557 selected villages during the months of February to April 2005. The second stage was performed by randomly selecting the sample of 50% households from the household list in each village.

All women aged 15 to 49 years who were either usual residents of the selected households or visitors present in the household on the night before the survey were eligible to be interviewed. The response rate was 98%.

Anthropometric indicators of children younger than 5 years of age were assessed in the randomly chosen 50% of the selected households [15].

### Main exposures

Food consumption was assessed for the youngest child in each household by asking his/her mother or caretaker if the child had eaten food from the following 16 food items within 24 hours before the survey. The 16 food items were (1) tinned/powdered or fresh milk; (2) baby formula; (3) commercial baby cereal; (4) porridge; (5) chicken, duck, or other fowls; (6) nuts; (7) liver, kidney, heart, or other organs; (8) bread, rice, noodles, or other grains; (9) white potato/yam, manioc, cassava, or other tubers; (10) eggs; (11) beef, pork, lamb, goat, rabbit, or deer; (12) pumpkin, carrots, squash, or sweet potatoes; (13) dark green leafy vegetables; (14) fish or shellfish; (15) beans, peas, or lentils; and (16) oil, fat, or butter.

We classified the 16 food items into seven food groups according to the WHO IYCF model chapter: (1) staple foods group included 4 items (bread, rice, noodles, or other grains; white potato/yam, manioc, cassava, or other tuber; commercial baby cereal; and porridge); (2) animal source foods group included 5 items (beef, pork, lamb, goat, rabbit or deer; chicken, duck or other birds; liver, kidney, heart or other organs; eggs; and fish or shellfish); (3) milk products group included 2 items (tinned/powder or fresh milk; and baby formula); (4) green leafy/orange color vegetables group included 2 items (dark green leafy vegetables; and pumpkin, carrots, squash, or sweet potato); (5) pulses group included 1 item (beans, peas, or lentils); (6) oils/fats group included 1 item (oils, fats, and butter); and (7) seeds group included 1 item (nuts) [12]. Consumption of each food group was defined as “yes” when the child had consumed at least one food item within the food group and “no” when the child had not consumed any food item within the food group.

Dietary diversity was evaluated based on the food variety score method [16]. In this study, the food variety score (FVS) ranged from zero to nine. When the sum of number of food items was less than 9, the sum was regarded as the score. When the sum of number of food items was nine or more, 9 was given as the score.

### Outcomes

Height was measured by using the measuring board made by Shorr Productions. Lying down (recumbent length) was carried out for children aged 0 to 23 months and standing height was measured for children aged 24 to 59 months. The unit of height measurement was 1 mm.

Weight was measured by using a lightweight electronic SECA scale according to the guidelines of the United Nations Children's Fund. For young child who could not stand up, the scale weighed him or her through an automatic mother-child adjustment that subtracted the mother’s weight while she was standing on the scale with her child. The unit of weight measurement was 100 g.

Height-for-age, weight-for-age, and weight-for-height were calculated according to the WHO Standard Reference 2006. The recommendation to use this reference population was based on the finding that well-nourished young children in all population groups (for which data exist) follow very similar growth patterns and thus exhibit similar distributions of height and weight at given ages [17].

The three indicators, height-for-age, weight-for-age, and weight-for-height, were expressed as “z-scores", which were defined as deviations from the median of the reference population in standard deviation units. These were used as nutritional status indicators of stunting, underweight, and wasting. Z-scores below −2 for height-for-age, weight-for-age and weight-for-height were defined as stunting, underweight, and wasting, respectively [17].

Any episode of diarrhea among children in the 2 weeks preceding the survey was reported by the mothers in response to specific questions.

### Covariates

We used socioeconomic and geographic variables including household wealth quintile, educational level of mother (no schooling, primary or below primary, and secondary or higher), geographical area (around the Tonlesap lake, coastal, plateau or mountain, and plain or Phnom Penh), and residential location (rural and urban) as potential confounders. The household wealth quintile was constructed by using a principle component analysis [18]. This indicator combined information on a set of household assets and living conditions including: telephone, television, radio, refrigerator, the ownership of a car, motorcycle, and bicycle; the availability of electricity, clean water and a toilet; and the material used to construct the wall, roof and floor of the household dwelling.

### Statistical analysis

We included children aged 12 to 59 months in the analysis.

The crude association between consumption of each food group and household wealth or education of mother was assessed by a chi-square test for trend, consumption of each food group and geographical area or residential location by a chi-square test, the FVS and household wealth or education of mother by a simple linear regression analysis, and the FVS and geographical area or residential location by a one-way analysis of variance.

The crude association between the health outcomes (stunting, underweight, wasting, and diarrhea) and household wealth or education of mother was assessed by a chi-square test for trend, and that between the health outcomes and geographical area or residential area by a chi-square test.

The odds ratio of the association between FVS and the health outcomes was calculated with a logistic regression analysis. The goodness-of-fit of the categorical model and the linear model of FVS was compared by a likelihood ratio test. The odds ratios were adjusted for wealth quintile, educational level of mother, geographic area, and residential location. We defined that the association was statistically significant when p < 0.05 in both a categorical model and a linear model.

We also evaluated the association between consumption of each food group and an outcome with a logistic regression analysis by adjusting socioeconomic and geographical factors.

Each piece of data was weighted by a sampling weight. The weight was the inverse of household selection probability multiplied by the inverse of the household response rate of its household response rate group and multiplied by the inverse of the individual response rate of his/her individual rate group.

### Ethical clearance

The interviewers of the CDHS received oral informed consent from the mothers of the children before beginning the interviews and taking anthropometric measurements. This study utilized publicly available anonymous data and was exempted from institutional ethical review.

## Results

Of the 6209 children aged 12 to 59 months, there were 4249 and 5707 children with complete information on food consumption and diarrhea, respectively. Of the 2783 children selected for anthropometric measurement, 2708 had information about both height and weight.

Table 1 shows the association between consumption of food groups, the FVS, and socioeconomic and geographic variables. Children who lived in a better-off household were more likely to consume staple foods, animal source foods, milk products, green leafy and orange color vegetables, pulses, and oils and fats than those from poorer households (all trend *P* < 0.01). Children whose mothers had attained higher levels of education were more likely to consume animal source foods, milk products, green leafy and orange color vegetables, pulses, and oils and fats than those whose mothers had received only lower levels of education (all trend *P* < 0.05). The percentage of children who consumed animal source food was highest among those who lived in the coastal area, and it was lowest among those in the plateau/mountain area. Children from the plains area were the most likely to consume milk products and pulses, but least likely to consume green leafy and orange color vegetables. Staple foods, animal source foods, milk products, and green leafy and orange color vegetables were more frequently consumed in urban areas than in rural (all *P* < 0.05). The average of FVS was positively associated with household wealth levels and educational level of the mothers (both *P* < 0.01). Children who lived in the coastal area had the highest average FVS, further, the average FVS was higher in urban than in rural area (both *P* < 0.01).

**Table 1:** Association between consumption of specific food groups, food variety score, and socioeconomic and geographic variables

**Socioeconomic and geographic variables**	**n**	**% consumed in the last 24 hours**							**Food variety score**	
		**Staple foods group**	**Animal source foods group**	**Milk products group**	**Green leafy/orange color vegetables group**	**Pulses group**	**Oils/fats group**	**Seeds group**	**Mean**	**SD**
Household wealth quintile										
Poorest	1233	86.9	74.8	1.4	47.9	4.4	19.1	5.2	3.3	2.0
Poorer	962	85.9	77.1	4.7	47.9	3.8	22.2	6.9	3.5	2.1
Middle	709	90.0	84.6	4.4	55.3	8.2	23.8	7.1	4.1	2.2
Richer	672	90.8	84.8	12.4	58.9	10.6	28.6	5.5	4.5	2.4
Richest	673	91.8	84.5	34.9	56.8	11.1	26.3	4.6	5.2	2.6
Trend P		<0.01	<0.01	<0.01	<0.01	<0.01	<0.01	0.57	<0.01	
Education of mother										
None	1045	87.8	78.2	4.1	50.1	4.2	20.4	6.4	3.5	2.1
Primary	2510	88.4	80.0	7.4	52.2	7.5	24.1	5.6	3.9	2.3
Secondary or higher	694	90.2	83.0	26.2	55.8	9.4	24.2	5.8	4.8	2.6
Trend P		0.15	0.02	<0.01	0.02	<0.01	0.04	0.50	<0.01	
Geographical area										
Near the Tolesap lake	1433	88.1	81.5	5.2	55.4	6.0	28.7	6.4	3.9	2.3
Coastal	336	89.3	82.4	12.5	62.2	5.0	14.2	6.3	4.3	2.2
Plateau or moutain	590	88.0	72.4	5.6	51.2	4.6	21.7	8.1	3.7	2.3
Plain or Phnom Penh	1889	88.9	80.9	13.8	48.4	8.8	21.1	4.6	4.0	2.3
P		0.83	<0.01	<0.01	<0.01	<0.01	<0.01	<0.01	<0.01	
Residential location										
Rural	3664	88.2	79.4	6.8	50.8	6.9	22.9	6.1	3.8	2.3
Urban	585	90.9	84.2	27.7	61.1	7.5	25.1	3.9	4.8	2.4
P		0.05	<0.01	<0.01	<0.01	0.57	0.24	0.04	<0.01	

The P value for the association between consumption of specific food group and a socioeconomic or a geographic variable was obtained by a chi-square test for trend (trend P) or by a chi-square test (P). The p value for the association between the food variety score and a socioeconomic or geographic variable was obtained by a linear regression analysis (trend P) or a one-way analysis of variance (P).

The prevalence of stunting, underweight, wasting, and diarrhea within 2 weeks were 48.5%, 31.2%, 7.3%, and 18.3%, respectively, among children aged 12 to 59 months.

Table 2 presents the association between socioeconomic characteristics and stunting, underweight, wasting, and diarrhea. Children who lived in better-off households were less likely to have stunting, underweight, and diarrhea than those who were from poorer families (*P* < 0.01, *P* < 0.01, and *P* = 0.01, respectively). Children whose mother had secondary education or higher were less likely to have stunting, underweight, and diarrhea than those whose mother had lower level of education (*P* < 0.01, *P* < 0.01, and *P* = 0.02, respectively). Children who lived in the plain or Phnom Penh area were less likely to have stunting (*P* < 0.01) but they were more likely to have diarrhea than those who lived in the other geographical areas (*P* < 0.01). Children who lived in urban areas were less likely to have stunting and diarrhea those who lived in rural areas (*P* < 0.01, and *P* = 0.04, respectively).

**Table 2:** Prevalence of stunting, underweight, wasting, and diarrhea by socioeconomic and geographic characteristics

**Socioeconomic and geographic variables**	**Stunting**		**Underweight**		**Wasting**		**Diarrhea**	
	**n**	**%**	**n**	**%**	**n**	**%**	**n**	**%**
Household wealth quintile								
Poorest	515	55.7	515	33.2	515	8.2	1151	22.2
Poorer	420	54.7	420	34.5	420	10.3	914	22.3
Middle	337	49.9	337	27.9	337	6.2	662	20.1
Richer	311	41.2	311	27.7	311	4.2	646	20.3
Richest	325	25.9	325	16.0	325	7.1	654	15.6
Trend P	<0.01		<0.01		0.06		<0.01	
Education of mother								
None	469	57.4	469	32.0	469	8.5	990	22.2
Primary	1106	47.8	1106	30.3	1106	7.3	2364	20.6
Secondary or higher	332	29.5	332	19.0	332	6.0	673	17.5
Trend P	<0.01		<0.01		0.18		0.02	
Geographical area								
Near the Tolesap lake	660	56.1	660	31.5	660	6.7	1360	19.2
Coastal	169	40.8	169	25.6	169	6.0	319	11.9
Plateau or moutain	263	51.0	263	31.6	263	8.4	552	20.3
Plain or Phnom Penh	815	39.6	815	26.3	815	8.0	1794	23.1
P	<0.01		0.08		0.62		<0.01	
Residential location								
Rural	1635	48.3	1635	29.0	1635	7.3	3466	21.0
Urban	272	39.3	272	27.2	272	8.5	561	17.3
P	<0.01		0.55		0.49		0.04	

The P value for the association between child nutritional status or diarrhea and a socioeconomic or a geographic variable was obtained by a chi-square test for trend (trend P) or by a chi-square test (P).

Table 3 presents the association between dietary diversity and stunting, underweight, wasting, and diarrhea after controlling for wealth index, educational level of mother, geographical and residential area. Based on the analysis using FVS as categorical variable, consumption of a diverse diet was significantly associated with stunting, and underweight (*P* value for categorical model =0.03, and 0.02, respectively) but it was not found to be statistically associated with wasting and diarrhea. Additional analysis using FVS as continuous variable showed that consumption of a diverse diet was significantly associated with reduction of stunting (OR_adj_ 0.95, 95% CI 0.91-0.99, *P* = 0.01) but it was not significantly associated with underweight. Therefore, both models of analysis showed that children who consumed diverse diets were less likely to be stunted than those who consumed less. The likelihood ratio test from the comparison of the goodness-of-fit of the categorical model and the linear model of FVS was not significantly different. In the further analysis, a linear model of FVS was used to assess the association between consumption of diverse foods and stunting, underweight, wasting, and diarrhea.

**Table 3:** Association between food variety score and stunting, underweight, wasting, and diarrhea

**Food variety score**	**n**	**Stunting**			**n**	**Underweight**			**n**	**Wasting**			**n**	**Diarrhea**		
		**%**	**Odds ratio**	**95% confidence interval**		**%**	**Odds ratio**	**95% confidence interval**		**%**	**Odds ratio**	**95% confidence interval**		**%**	**Odds ratio**	**95% confidence interval**
Categorical model																
0	171	53.2	Reference		171	32.2	Reference		171	7.1	Reference		394	18.0	Reference	
1	87	59.8	1.33	0.77-2.29	87	37.9	1.37	0.79-2.37	87	6.9	1.37	0.79-2.37	201	16.4	0.92	0.58-1.46
2	157	58.6	1.16	0.74-1.82	157	34.4	1.05	0.66-1.67	157	9.0	1.05	0.66-1.67	335	21.8	1.20	0.83-1.73
3	333	48.8	0.82	0.56-1.20	333	29.1	0.85	0.56-1.27	333	6.0	0.85	0.56-1.27	697	19.9	1.12	0.81-1.54
4	340	43.8	0.71	0.48-1.04	340	25.3	0.73	0.49-1.10	340	6.7	0.73	0.49-1.10	778	22.6	1.33	0.98-1.82
5	343	46.8	0.90	0.61-1.32	343	31.2	1.05	0.70-1.58	343	7.3	1.05	0.70-1.58	657	22.2	1.39	1.01-1.91
6	203	43.3	0.88	0.57-1.35	203	32.0	1.21	0.77-1.90	203	11.3	1.21	0.77-1.90	456	20.2	1.39	0.96-1.97
7	140	45.0	0.94	0.59-1.50	140	20.7	0.67	0.39-1.14	140	4.3	0.67	0.39-1.14	260	19.6	1.30	0.87-1.95
8	63	25.4	0.43	0.22-0.83	63	17.5	0.59	0.28-1.22	63	9.5	0.59	0.28-1.22	120	16.7	1.06	0.61-1.85
9a	70	31.4	0.58	0.31-1.09	70	15.7	0.52	0.25-1.08	70	7.2	0.52	0.25-1.08	126	19.0	1.36	0.80-2.30
			P = 0.03				P = 0.02				P = 0.32				P = 0.38	
Linear model																
per 1 FVS			0.95	0.91-0.99			0.96	0.91-1.00			0.96	0.91-1.00			1.04	1.00-1.08
			trend P = 0.01				trend P = 0.06				trend P = 0.24				trend P = 0.03	

The odds ratio was adjusted for household wealth quintile, education of mother, geographical area and residential location. The same 10 categories were used in linear models as in categorical models. Categorical models and linear models fit the data equally well (all P > 0.10 in likelihood ratio test for categorical vs. linear model).

a Food variety score of nine or higher.

Table 4 shows the association between consumption of specific food groups and stunting, underweight, wasting, and diarrhea. Children who consumed animal source food were less likely to be stunting and underweight (OR_adj_ 0.69, 95% CI 0.54-0.89, *P* < 0.01, and OR_adj_ 0.74, 95% CI 0.57-0.96, *P* = 0.03, respectively). On the contrary, the higher risk of diarrhea was significantly associated with consumption of milk products (OR_adj_ 1.46, 95% CI 1.01-1.92, *P* = 0.02), after controlling for socioeconomic and geographical variables. This relationship between consumption of milk products and diarrhea were further analyzed by household wealth by adjusting other variables. Significant association was found among the poorest, poorer, or middle households (OR_adj_ 1.85, 95% CI 1.17-2.93, *P <* 0.01) but it was not found among the richer or the richest households (OR_adj_ 1.15, 95% CI 0.81-1.63, *P* = 0.42).

**Table 4:** Association between consumption of specific food groups and stunting, underweight, wasting, and diarrhea

**Food group**	**n**	**Stunting**			**n**	**Underweight,%**			**n**	**Wasting**			**n**	**Diarrhea**		
		**%**	**Odds ratio**	**95% confidence interval**		**%**	**Odds ratio**	**95% confidence interval**		**%**	**Odds ratio**	**95% confidence interval**		**%**	**Odds ratio**	**95% confidence interval**
Staple foods group																
No	190	54.2	reference		190	32.1	reference		190	7.3	reference		438	17.4	reference	
Yes	1717	46.2	0.80	0.59-1.09	1717	28.3	0.88	0.63-1.22	1717	7.5	1.10	0.61-2.00	3588	20.9	1.29	0.99-1.67
Animal source foods group																
No	329	56.5	reference		329	35.2	reference		329	6.7	reference		774	18.6	reference	
Yes	1578	45.0	0.69	0.54-0.89**	1578	27.4	0.74	0.57-0.96*	1578	7.5	1.22	0.76-0.1.97	3252	20.8	1.20	0.98-1.47
Milk products group																
No	1713	48.3	reference		1713	29.4	reference		1713	7.7	reference		3628	20.2	reference	
Yes	193	35.2	1.02	0.72-1.44	193	22.8	1.02	0.70-1.49	193	4.7	0.61	0.29-1.26	399	21.9	1.46	1.10-1.92*
Green leafy/orange color vegetables group																
No	873	49.0	reference		873	30.2	reference		873	7.2	reference		1896	20.8	reference	
Yes	1034	45.2	0.94	0.78-1.13	1034	27.3	0.94	0.76-1.15	1034	7.6	1.18	0.83-1.68	2130	20.1	1.02	0.87-1.19
Pulses group																
No	1767	47.7	reference		1767	29.1	reference		1767	7.2	reference		3735	20.3	reference	
Yes	140	37.9	0.84	0.58-1.22	140	23.6	0.88	0.59-1.33	140	10.1	1.74	0.96-3.15	291	21.9	1.13	0.85-1.51
Oils/fats group																
No	1432	47.6	reference		1432	29.3	reference		1432	7.5	reference		3081	20.3	reference	
Yes	476	44.9	0.87	0.7-1.08	476	26.8	0.87	0.69-1.11	476	7.4	1.04	0.69-1.55	946	20.9	1.08	0.90-1.29
Seeds group																
No	1782	46.7	reference		1782	28.8	reference		1782	7.5	reference		3788	20.4	reference	
Yes	124	50.8	1.15	0.78-1.67	124	27.0	0.92	0.61-1.39	124	6.4	0.83	0.39-1.75	239	20.2	1.01	0.73-1.40

Odds ratio was obtained by a logistic regression analysis and adjusted for household wealth quintile, education of mother, geographical area and residential location.

*P < 0.05, **P < 0.01.

The average score of FVS among children who consumed the animal source food group was higher than those who did not consumed it (4.7 ± 1.8 versus 0.9 ± 1.2, *P* <0.01).

## Discussion

This study examined the relation between dietary diversity, consumption of specific food groups, and stunting, underweight, and diarrhea among children aged 12 to 59 months. We found that a diverse diet was a protective factor of stunting, but not of underweight. Consumption of animal source foods appeared to be independently associated with a decreased risk of stunting and underweight. A higher risk of diarrhea was identified among children consuming milk products.

Our study showed the benefit of a diverse diet on the height-for-age measurements among Cambodian children. A study in Bangladesh reported a negative association between dietary diversity and stunting after controlling for covariates [19]. In addition to evaluation in Asian countries, the association between consuming a diverse diet and stunting has been highlighted by empirical studies in Africa. A study in Burkina Faso reported that the dietary diversity was positively associated with height-for-age z score [20]. In a study in Ethiopia, dietary diversity that was measured on the basis of 24-hour recall of food intake was significantly associated with lower risk of stunting among children after controlling for child, maternal, and household socioeconomic factors [21].

Consumption of animal source foods was found to be associated with a decreased risk of stunting and underweight. A study that was conducted by Dror and Allen reported that consuming animal source foods not only decreased stunting but also improved other anthropometric indices toward the reduction of morbidity and mortality of undernourished children [22]. Children whose mothers consumed fish (classified as an animal source food) during pregnancy and lactation had significantly higher weight-for-age [23]. Animal source food was found to have a variety of micronutrients including vitamin A, vitamin B-12, riboflavin, calcium, iron and zinc that are difficult to obtain in adequate quantities from plant source foods alone [24]. These micronutrients enriched the type I and particularly type II nutrients (the growth nutrients) which are the building blocks of tissue and are important for biochemical pathway. When the body has insufficient intake of these nutrients, a child will not physically develop [25]. In addition, having a lack of or a short supply of these micronutrients was associated with a negative impact on health outcomes, such as poor growth, impaired cognitive performance, and death [26]. Thus animal source foods are good sources of nutrients that are required for growth, and also of micronutrients that support the immune system. Suggesting to parents and caregivers that they should improve child-feeding practices with diverse diet and animal source foods is a critical public health intervention in Cambodia, as well as in other developing countries.

We found that children who consumed milk products such as tinned, powdered or fresh milk and baby formula had a higher risk of diarrhea. Milk products are considered to be one of the basic food groups that provide proper nutrition to promote child health [27]. Many people are, however, less aware of the potential risk of diarrhea that is related to consuming milk products made unsafe by poor storage or pasteurization techniques. For instance, a study conducted in Mali found that intake of milk products increased the risk of food-borne toxic infections [28]. Low personal hygiene and sanitation of parents may contribute increasing of diarrhea to their children. The CDHS reported that about 40% of rural households and 20% of urban households drink water that has not been boiled or treated [15]. This study also showed the increased risk of diarrhea associated with consumption of milk product among children from the poorer households but there was no increase of risk among children from richer households. This indicates an interaction by household wealth for the risk of diarrhea according to the consumption of milk product. The mass communication for behavioral change campaign should educate parents and child caregivers not only about the health benefit of complementary foods, but also about the importance and practice of preparing safe milk for their children, particularly in communities with deprived socioeconomic conditions.

Other potential factors such as socioeconomic status are considered as proxy determinants of food security or adequacy of dietary intake that affects the nutritional status of children [29,30]. In this study, we found that food consumption and dietary diversity were positively associated with a better household economy, a higher educational level for the mother, and an urban location. The frequency of consumption of specific types of food varies by geographic area. A study conducted in Peru found that children whose mothers completed secondary or higher education were less likely to have stunting than those whose mothers had received no education [31]. Furthermore, household food insecurity, which was also associated with less educated mothers, was related to malnourished children [32]. Poverty in either urban or rural areas was a contributing risk factor of food insecurity [9]. Another study conducted in the Democratic Republic of Congo determined that the proportion of chronically malnourished children was markedly higher in rural areas than in urban areas, and the spatial distribution differed by geographical area [33].

The national consumer price index (CPI) indicated an upward trend of food prices since 2000. In depth investigation reported the rice price during raining seasons (August to November) between 2000 and 2005 ranged from 1,000 to 1,500 Riel/kg, while that before 2000 ranged from 600 to 900 Riel/kg [34]. It is reported that the intake of high-priced food, including eggs and meats was trimmed down particularly among poor households, in relation to this increase of rise price [34]. Among the better-off households, the expenditures on food do not change much by year. The better-off usually purchase fish, pork, eggs, vegetables, oil, sugar, and fruits, while the poor households’ expenditure is mostly for rice, vegetables, and fish [35]. The increase of food price not only influenced on the decline of consumption of food but also influenced on the reduction of households’ purchasing power, resulting in reduced utilization of health and welfare services which protect health [36,37]. A literature review on impact of the global food crisis on the poor found that the elevated food price not only increased risk of malnutrition but also poverty [38]. A study conducted in Antioquia, Colombia, assessing the association between household food insecurity and stunting and underweight among preschool children reported a dose–response relationship between food insecurity and child stunting and underweight [39]. When food diversity increase is limited by financial constraints, income transfers for the poor and provision of fortifying specific foods to particular groups are examples of promotion and facilitation of food diversity and consumption of animal source foods.

Traditional cultural practices among rural people and the current inflation in food prices resulted in low frequency of consumption of animal source foods in Cambodia. Chicken or ducks were kept in many households; however, they did not consume them often, because they usually sold them for income [40]. Long-term planning to change community behavior and practice toward consuming animal source foods through the most effective health education means possible is critical. Health education message related to child-feeding practices with a diverse diet and animal source foods is a critical public-health intervention in Cambodia, as well as in other developing countries. Mass communication of a behavioral-change campaign should include the importance and practice of preparing safe milk for children, particularly in communities with deprived socioeconomic conditions. Also, strong governmental commitment to help control the inflation of food prices should be considered, in order to strengthen the food security within any community.

Due to the nature of the cross-sectional study design, our results support the association between the food consumption and nutrition status of children, but do not prove the causal relationship. The information related to food consumption, which was obtained from respondents, has potential source of recall bias, but since DHS collected information on food consumption within 24 hours before the survey, the magnitude of bias is limited. Future studies which directly measure food items consumed by the subjects among Cambodia children would add detail information about nutritional intake, which enables nutritional experts to directly examine food consumption of large number of children in both urban and rural areas.

## Conclusions

This study showed that consumption of a diverse diet was associated with a reduction in stunting. In addition to dietary diversity, animal source food was a protective factor of stunting and underweight. Consumption of milk products was associated with an increase in the risk of diarrhea, particularly among the poorer households. Measures to meet nutrient needs of children particularly among those in socioeconomically deprived families should be considered. Strong commitment by the government to secure food for families, control food price, health education for child-feeding is required.

## Competing interests

The authors declare that they have no competing interests.

## Authors’ contributions

CD, MK, KN and KS designed the study. CD obtained and analyzed the data, and interpreted the results of analysis, and drafted an initial manuscript. MK, KN and KS participated in interpreting the data and developing the manuscript. TT supervised the study concept, analysis, and writing. All authors approved the final manuscript.
